# Protective role of extracellular vesicles against oxidative DNA damage

**DOI:** 10.1186/s40659-025-00595-5

**Published:** 2025-03-13

**Authors:** Jordi Ribas-Maynou, Ana Parra, Pablo Martínez-Díaz, Camila Peres Rubio, Xiomara Lucas, Marc Yeste, Jordi Roca, Isabel Barranco

**Affiliations:** 1 Department of Medicine and Animal Surgery, Faculty of Veterinary Science, University of Murcia, Murcia, Spain; 2 International Excellence Campus for Higher Education and Research “Campus Mare Nostrum”, Institute for Biomedical Research of Murcia (IMIB-Arrixaca), University of Murcia, Murcia, Spain; 3 Unit of Cell Biology and Medical Genetics; Department of Cell Biology, Physiology and Immunology, Autonomous University of Barcelona, Bellaterra, Spain; 4 Biotechnology of Animal and Human Reproduction (Technosperm), Institute of Food and Agricultural Technology, University of Girona, Girona, Spain; 5 Unit of Cell Biology, Department of Biology, Faculty of Sciences, University of Girona, Girona, Spain; 6 Catalan Institution for Research and Advanced Studies (ICREA), Barcelona, Spain

**Keywords:** DNA oxidation, DNA fragmentation, Extracellular vesicles, Peripheral corona layer, Seminal plasma, Single-strand breaks

## Abstract

**Background:**

Oxidative stress, a source of genotoxic damage, is often the underlying mechanism in many functional cell disorders. Extracellular vesicles (EVs) have been shown to be key regulators of cellular processes and may be involved in maintaining cellular redox balance. Herein, we aimed to develop a method to assess the effects of EVs on DNA oxidation using porcine seminal plasma extracellular vesicles (sEVs).

**Results:**

The technique was set using a cell-free plasmid DNA to avoid the bias generated by the uptake of sEVs by sperm cells, employing increasing concentrations of hydrogen peroxide (H_2_O_2_) that generate DNA single-strand breaks (SSBs). Because SSBs contain a free 3’-OH end that allow the extension through quantitative PCR, such extension -and therefore the SYBR intensity- showed to be proportional to the amount of SSB. In the next stage, H_2_O_2_ was co-incubated with two size-differentiated subpopulations (small and large) of permeabilized and non-permeabilized sEVs to assess whether the intravesicular content (IC) or the surface of sEVs protects the DNA from oxidative damage. Results obtained showed that the surface of small sEVs reduced the incidence of DNA SSBs (*P* < 0.05), whereas that of large sEVs had no impact on the generation of SSBs (*P* > 0.05). The IC showed no protective effect against DNA oxidation (*P* > 0.05).

**Conclusions:**

Our results suggest that the surface of small sEVs, including the peripheral corona layer, may exert a protective function against alterations that are originated by oxidative mechanisms. In addition, our in vitro study opens path to detect, localize and quantify the protective effects against oxidation of extracellular vesicles derived from different fluids, elucidating their function in physiopathological states.

**Supplementary Information:**

The online version contains supplementary material available at 10.1186/s40659-025-00595-5.

## Introduction

Extracellular vesicles (EVs) are membranous particles ranging in size from 50 to 1000 nm and are secreted by virtually all functional body cells [1]. EVs have been isolated from several body fluids and play an essential role in cell-to-cell communication [2]. Extensive research has shown that EVs are critically involved in several physiological cellular processes [3], contribute to disease dissemination [4], and may be useful as drug-delivery systems [5]. The EV population in body fluids is heterogeneous in size, as it includes exosomes (smaller than 200 nm) and ectosomes (50 to 1000 nm), which derive from endosomes and plasma membrane, respectively [1]. The mechanisms by which EVs exert their biological functions have been the subject of many studies, and their functionality has been shown to be related to the diverse cargo of proteins, lipids, and nucleic acids, amongst others [6–8]. While most of the cargo of EVs is intravesicular, mounting evidence supports that they may also carry extravesicular content [9, 10]. This extravesicular content, referred to as the content located in the EV surface, consists of an array of molecules that are either externally integrated or peripherally coupled to the EV membrane [11]. Then, not only the intravesicular content (IC) but also the composition of the EV surface must be considered when defining the functions of EVs. This knowledge is essential to evaluate the usefulness of EVs as therapeutic vehicles and to assess them as biomarkers of a healthy state [5, 12–14].

Oxidative stress (OS) and nitrosative stress (NS)—defined as an imbalance between prooxidant and antioxidant molecules—play an important role in the etiology of a wide range of diseases, such as cardiovascular, neurodegenerative, renal, cancer, autoimmune, or infertility, amongst others, and thus have severe implications for human and animal health [15–18]. Yet, although reactive oxygen species (ROS) and reactive nitrogen species (RNS) are by-products of normal metabolism, they can have beneficial roles in cellular physiology when present at low or moderate concentrations [19]. For instance, ROS and RNS can be involved in the protection against infectious agents and in several signaling pathways, and can act as mediators of mitogenic growth [20–22]. The maintenance of these physiological and controlled levels of prooxidant species, however, depends on the presence of antioxidants, which are molecules that scavenge the excess of ROS and RNS produced in response to OS and NS, these may be present in the cell cytoplasm and/or in the fluid surrounding the cells [23, 24]. When the oxidant/antioxidant system is imbalanced in favor of the former, OS and NS harm biological structures, causing modifications in proteins, lipids and nucleic acids, ultimately leading to the loss of normal cell function [25–29]. Regarding the effects on nucleic acids, oxidation and nitration of nitrogenous bases promote the formation of modified adducts, such as 8-oxo-7,8-dihydro-2’-deoxyguanosine (8-oxodG) and 8-nitroguanine, which necessarily need to be processed by the DNA repair systems, mainly the base excision repair pathway [30]. If, nevertheless, these genotoxic insults are not adequately repaired, they can lead to DNA single-strand breaks (SSBs), which can block DNA replication forks, inactivate RNA polymerases during transcription, increase genetic instability, generate double-stranded breaks and induce apoptosis [31, 32].

Recent reports proved that the EVs isolated from different body fluids or cell types may carry antioxidant components and/or signaling molecules that activate the antioxidant defenses in cells [33–35]. Oxidative stress maintenance is very important for sperm cell function, and ROS are the main molecules impairing sperm function. Therefore, the presence of intracellular and extracellular antioxidants is key for the preservation of sperm [36]. Specifically, for seminal plasma, several antioxidant enzymes and molecules with antioxidant properties have been identified in EVs [37]. Their presence has directly been associated to the regulation of oxidative stress by increasing the sperm total antioxidant capacity [38], being also involved in sperm function by controlling metabolism and ultimately being related to fertilization ability [39, 40]. To the best of our knowledge, however, no previous report has interrogated the protective effect of EVs on the generation of SSBs. To this end, the main objective of the present study was to develop a specific method to evaluate the protective capacity of EVs against DNA oxidation, which ultimately generates SSBs, using a cell-free system without the fusion of seminal EVs (sEVs) with sperm cells. The use of porcine seminal plasma as a model was motivated by the fact that the pig is an excellent animal model for human health [41], and that its seminal plasma is a particularly EV-rich fluid [42].

## Materials and methods

### Seminal plasma samples and bioethical aspects

The experiments were approved by the Bioethics Committee of the University of Murcia in its meetings of March 25, 2021, and June 16, 2023, with research codes CBE 367/2020 and CBE 538/2023, respectively. The present study used boar semen samples that were provided by an artificial insemination (AI) center (AIM Iberica, Topigs Norsvin Spain SLU; Calasparra, Murcia, Spain), which complies with the European (ES13RS04P, July 2012) and Spanish (ES300130640127, August 2006) regulations for the commercialization of AI semen doses, and animal health and welfare.

Ejaculate donors were 24 healthy, mature and fertile Landrace × Large White boars regularly used as semen donors in commercial AI programs. The ejaculates met the quality criteria for the production of commercial doses of AI semen (i.e., > 200 × 10^6^ spermatozoa/mL, > 70% motile spermatozoa and > 75% morphologically normal spermatozoa).

Entire ejaculates (n = 24; one ejaculate per boar) were collected using a semi-automated method (Collectis^®^, IMV Technologies; L’Aigle, France). Immediately after collection, 15 mL of each ejaculate was centrifuged twice at 3000 × g at room temperature (RT) for 10 min. The supernatant (seminal plasma) was treated with a protease inhibitor cocktail (Roche complete™ Protease Inhibitor Cocktail tablets; Basel, Switzerland) and stored at 5 °C until used for sEV isolation (max. 12 h after ejaculate collection).

### Plasmid DNA preparation

The plasmid used for our in vitro experiments was pGADT7-T (Takara Bio; Kusatsu, Japan), whose length was 9973 bp. To obtain the necessary amount of plasmid, it was transformed into a commercial bacterial system (*E. coli* One Shot TOP10, Thermo Fisher Scientific, Whaltham, MA, USA), which was allowed to grow. Plasmid was purified through the Qiagen Plasmid Midi Kit (Qiagen; Hilden, Germany), quantified and checked for DNA integrity.

#### Plasmid transformation, purification, quantification and evaluation of DNA integrity

The pGADT7-T plasmid was transformed into *E. coli* One Shot TOP10 competent cells (Thermo Fisher Scientific catalog #C404003; Waltham, MA, USA) under sterile conditions following the manufacturer’s instructions. After the transformation procedure, the transformants were plated onto a petri dish containing Luria-Betani (LB) agar supplemented with 100 µg/µL ampicillin, and incubated at 37 °C overnight. Among the grown bacteria, one colony was picked using a loop handle and was inoculated in 5 mL LB broth containing 100 µg/µL ampicillin, allowed to grow for 8 h at 37 °C. Then, a 100 mL culture was grown in the same media. Sterility controls were conducted at each step.

Plasmid purification was performed using the commercial Qiagen Plasmid Midi Kit (Qiagen) according to the manufacturer’s instructions for 100 mL of bacterial culture. After the standard procedure, the resulting pellet was air-dried and resuspended in 100 µL of diethylpyrocarbonate (DEPC)-treated water (RNase-free water), aliquoted, and stored at − 80 °C until further use.

Plasmid quantification and quality assessment (260/280 nm absorbance ratio) were performed by spectrophotometry (Agilent Epoch spectrophotometer, Metler Toledo; Giessen, Germany). Plasmid DNA integrity was checked by electrophoresis on a 2% agarose gel containing 1 × SYBR-safe DNA stain (Thermo Fisher Scientific Whaltham, MA, USA), which was run at 60 V for 1 h at 4 °C in Tris–EDTA (TE) buffer. The gel was visualized using a G:Box Chemi XL 1.4 (SynGene; Frederick, MT, USA) system.

### Evaluation of oxidative DNA damage in cell-free DNA

To establish a methodology to assess and quantify the protective activity of sEVs against oxidative DNA damage, we developed a procedure based on detecting the SSBs generated by the oxidation of nitrogenous bases using a modified quantitative PCR (qPCR) without primers. To reduce the bias caused by cellular factors and iatrogenic generation of DNA nicks during genomic DNA extraction and manipulation, we established this technique in a cell-free plasmid DNA, produced as described above in Sect. “Plasmid DNA preparation”. This subsection will detail the generation of SSB, the evaluation of DNA breaks through qPCR-based method, and its validation through Terminal dUTP Nick End Labeling assay.

#### Generation of single-strand DNA breaks as positive controls

To validate the DNA damage detection method and evaluate the protective effect of antioxidant compounds, positive controls containing increasing amounts of ROS-induced DNA SSBs were generated. For this purpose, 1 µg of pGADT7-T plasmid was incubated with different concentrations of H_2_O_2_ (0 mM, 0.0001 mM, 0.001 mM, 0.01 mM, 0.1 mM, 1 mM) at 37 °C for 5 min. The reaction was stopped by adding 3.5 units of catalase (Sigma Aldrich, catalog #C1345; St. Louis, MI, USA), and the mixture was subsequently incubated at 37 °C for 5 min. The catalase was then inactivated by heat at 60 °C for 10 min. Reactive oxygen species are known to oxidize nitrogenous bases, which are actively cleaved in living cells to generate SSBs thanks to the catalytic activities of DNA glycosylase and apurinic or apyrimidinic lyase (AP lyase). In our system, we included a final incubation step with 1 unit of formamidopyrimidine DNA glycosylase (FPG, New England Biolabs; Ipswich, MA, USA), which possesses these catalytic activities, at 37 °C for 1 h. This incubation thus released damaged purines like 8-oxodG and generated abasic sites with a free 3’-OH end in the plasmid. After incubation, FPG was inactivated by heating at 60 °C for 10 min.

#### qPCR-based evaluation of DNA single strand breaks

**Fig. 1:**
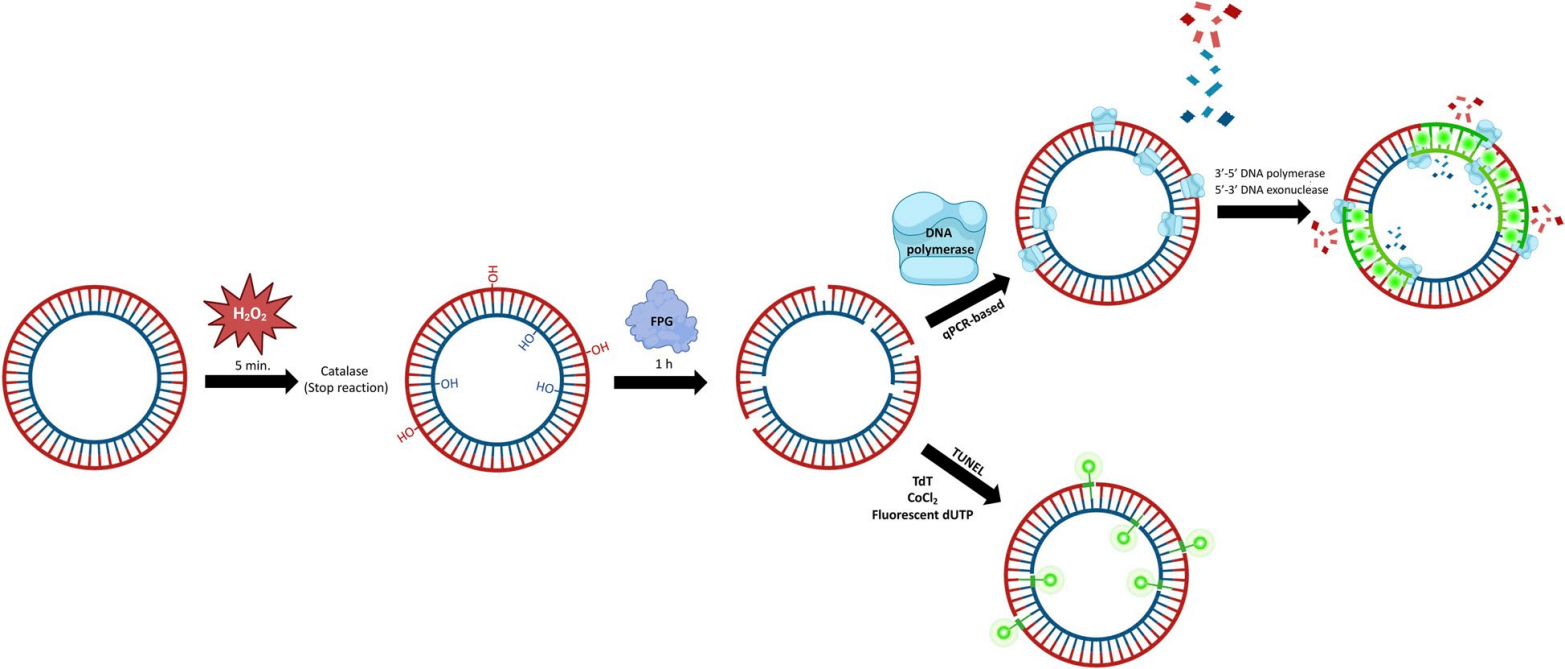
Schematic overview of the procedure conducted to generate oxidative DNA single-strand breaks, and their detection through a quantitative PCR-based method and a TUNEL assay. First, a treatment with hydrogen peroxide (H_2_O_2_) causes oxidation of nitrogenous bases, which are excised by the formamidopyrimidine DNA glycosylase (FPG) enzyme. The generated nicks can serve as priming sites for the quantitative PCR-based method and can be detected with the TUNEL assay

The DNA containing SSBs was detected by a modified qPCR in which no forward and reverse primers were added. The free 3’-OH ends generated at the abasic sites were used as priming sites for DNA polymerase. Briefly, 325 ng of test DNA was co-incubated with 10 µL of SYBR select Master Mix (Thermo Fisher Scientific, catalog #4472908), which contains an AmpliTaq DNA polymerase with 5’−3’ exonuclease activity, in a total volume of 20 µL adjusted with DEPC water. The real-time thermocycler 7500 Real Time PCR system (Applied Biosystems; Waltham, MA, USA) was used to acquire the fluorescence at the FITC channel. The incubation conditions were: 1 min at 25 °C followed by 60 min at 72 °C, which allowed for the annealing and extension by the DNA polymerase. The SYBR fluorescence and the passive reference fluorescence (ROX) were acquired every minute, and the mean fluorescence intensity value was recorded between minutes 10 and 40. The ratio between SYBR/ROX was used as an indicator of DNA replication. Two technical replicates were performed for each measurement, and a negative control without DNA was included to determine the background fluorescence. A schematic overview of the procedure is shown in Fig. 1.

To determine the amount of DNA required for the qPCR reaction, an experiment consisting of the protocols described herein was conducted, but on this occasion we used four different amounts of test DNA: 900 ng, 650 ng, 325 ng and 165 ng.

#### Terminal d-UTP nick end labeling (TUNEL) validation

To validate our qPCR setup, a modified TUNEL assay was performed using the In Situ Cell Death Detection Kit, Fluorescein (Roche catalog #11684795910; Basel, Switzerland). Briefly, samples containing 1 µg of plasmid DNA in a total volume of 10 µL were mixed with 5 µL of TUNEL enzyme and 35 µL of labeling solution, and incubated at 37 °C in the dark for 60 min. The DNA was then purified using the QIAquick PCR Purification Kit (Qiagen; Hilden, Germany), which is used here to remove enzymes, free nucleotides, and small DNA fragments from the final solution. This step was essential because dUTPs bound to the free 3’-OH ends and soluble dUTPs emit fluorescence, which can interfere with the result. The PCR purification kit was used following the manufacturer’s instructions, at RT in the dark. The final product was evaluated for fluorescence intensity in three independent measurements. The 7500-thermocycler real-time PCR system (Applied Biosystems; Waltham, MA, USA) was set to detect fluorescence in the FITC channel, maintaining the temperature at 25 °C. A control measurement of the fluorescence intensity of DEPC water was performed as a negative control to subtract the basal fluorescence of the tested samples. A diagram of how the TUNEL assay was used to detect the SSBs present in plasmid DNA is shown in Fig. 1.

### Isolation and characterization of extracellular vesicles

#### Isolation of extracellular vesicles

Seminal EVs (sEVs) were isolated from six seminal plasma samples. Each sample was a mixture of four seminal plasma samples, each from a different ejaculate. The isolation method was based on size exclusion chromatography (SEC), as described in detail by Barranco et al. [39]. Briefly, seminal plasma samples free of cellular debris (4 mL) were centrifuged at 20,000 × g and 4 °C for 30 min (Sorvall™ Legend™ Micro 21R, Thermo Fisher Scientific) and the resulting pellets and supernatants were separately subjected to SEC. The pellets were used to isolate large sEVs and the supernatants were used to isolate small sEVs. The SEC columns consisted of home-made filtration tubes (Econo-Pac^®^ Chromatography Columns, Bio-Rad, Hercules, California, USA) loaded with Sepharose CL2B^®^ (10 mL, Merck, Darmstadt, Germany). From each SEC, the eluted fractions 7 to 10, out of a total of 20, were selected as being the richest in sEVs and were mixed to obtain a single 2 mL sample. A 0.5 mL aliquot was used for sEV characterization. The remaining 1.5 mL were ultrafiltered using a 2 mL MWCO 100 kDa ultrafilter (Amicon^®^ Merck) and a centrifugation cycle of 4000 × g for 45 min at 4 °C to concentrate de sEVs. The concentrated sEV samples were stored at − 80 °C (Ultra Low Freezer; Haier Inc., Qingdao, China) until used in the experiment.

#### Characterization of extracellular vesicles

The sEV samples were characterized following the guidelines of the Minimum Information for Extracellular Vesicle Studies (MISEV2023; [43]). Characterization included the assessment of (1) total protein concentration, (2) particle size distribution, (3) morphology of sEVs, (4) EV-specific protein markers, and (5) markers of non-vesicular extracellular particles. Total protein concentration was quantified by measuring the absorbance at 280 nm using a Nanodrop 200 device (Thermo Fisher Scientific). Particle size distributions were assessed by dynamic light scattering (DLS) analysis using a Zetasizer Nano ZS system (Malvern Panalytical, Malvern, UK). The morphology of sEVs was examined by cryo-electron microscopy (cryo-EM) using a JEM-2200FS/CR electron microscope (JEOL, Tokyo, Japan) according to the protocol described by Parra et al. [44]. The presence of the two EV-specific protein markers CD81 and HSP70/HSC70 and the non-vesicular extracellular particle marker albumin was assessed using a high-sensitivity flow cytometer (CytoFLEX S, Beckman Coulter, Life Sciences Division Headquarters,Indianapolis, USA), according to the protocol described by Barranco et al. [39], which included labeling of sEVs with carboxyfluorescein succinimidyl ester (CFSE,CellTrace ™, Thermo Fisher Scientific) prior to analysis of protein EV markers. The antibodies used were anti-CD81-APC (130-119-787, Miltenyi Biotec, Pozuelo de Alarcón, Madrid, Spain), anti-HSP70/HSC70-APC (N27F3-4, Invitrogen™, Waltham, MA, USA), and anti-swine albumin-FITC, (CLFAG16140, Cedarlane, Burlington, VT, USA).

### Protective activity of extracellular vesicles against oxidative DNA damage

#### Determination of the amount of sEVs needed in the cell-free system to mimic biological conditions

Total protein concentration was used as a method to indirectly measure the sEVs amount in each sEV sample. However, because the protocols described above to evaluate DNA damage were applied to cell-free DNA (soluble plasmid DNA), no reference values for the number of sEVs per µg of DNA were known. For this reason, we conducted an experiment to establish the equivalence between the sperm count and their DNA content. Different amounts of pig sperm (0.5 × 10^6^, 3 × 10^6^, 5 × 10^6^, and 10 × 10^6^ spermatozoa) were centrifuged at 2000 × g, and the supernatants were discarded. Then, the total DNA was extracted using the blood and tissue DNA extraction kit (Qiagen), following the manufacturer’s instructions. Two technical replicates were examined, and a negative control containing only the AE buffer was included to subtract the basal value from the DNA values. After a regression analysis, a formula relating the number of spermatozoa to the amount of DNA extracted was worked out. This formula was used to extrapolate the number of sEVs needed for the subsequent experiments, which aimed to compare the antioxidant capacity of the IC and the EV surface between the two sEV subtype samples, namely small and large.

#### Co-incubation of sEVs and ROS to determine the protective capacity of sEVs against oxidative DNA damage

A previous study in pigs found that sEVs affect sperm functionality at a concentration of 0.1 mg/mL protein per 1 × 10^6^ sperm/mL [40]. A total of 1.43 mg of sEVs protein per µg of DNA and mL of experiment was used, considering that, according to our previous data, 14.3 × 10^6^ sperm corresponded to 1 µg of DNA. Since the experiments were performed in a volume of 10 µL and contained 1 µg of plasmid DNA, the total amount of sEV was equivalent to 14.3 µg of protein for each experimental condition.

The protective capacity of sEVs against oxidative DNA damage was analyzed using samples of small and large sEVs that underwent or did not undergo permeabilization. This approach aimed to address whether the protective capacity of sEVs resided in the IC or the EV surface. For vesicle permeabilization, sEV samples were incubated with 0.1% Triton X-100 in Phosphate-buffered saline (PBS, Merck) at RT for 30 min. Then, permeabilized and non-permeabilized small or large sEV samples were co-incubated with 1 µg pGADT7-T plasmid and H_2_O_2_ at 37 °C for 5 min. The tested concentrations of H_2_O_2_ were those that produced oxidative DNA damage (Sect. “Generation of single-strand DNA breaks as positive controls”). Catalase (3.5 units) was used to stop the reaction, and FPG enzyme was utilized to excise the damaged bases. The SSBs were quantified employing the qPCR-based protocol described in Sect. “qPCR-based evaluation of DNA single strand breaks”, which uses the SYBR/ROX ratio as an indicator. Twelve sEVs samples (six small and six large sEV samples) isolated from the six pools were used, and two technical replicates for each of the tested conditions were performed.

### Determination of the total antioxidant capacity of sEVs

The antioxidant capacity of sEV samples was based on the analysis of cupric ion reducing antioxidant capacity (CUPRAC) and thiol-reactive antioxidant molecules (THIOL). The CUPRAC assay measures non-enzymatic antioxidants, and the THIOL assay measures enzymatic antioxidants. Assays were performed on non-permeabilized and permeabilized sEV samples. For permeabilization, 25 μL of sEV sample was mixed with 25 μL of lysis solution containing 0.1% Triton X-100 in PBS (Merck), and the mixture was incubated at 37 °C with constant shaking (300 rpm) for 1 h.

The CUPRAC assay is based on the reduction of Cu^2+^ to Cu^+^ by the non-enzymatic antioxidants present in the sample [45]. The assay was performed following the protocol described by Banihani and Alawneh [46] with slight modifications. Briefly, 1 mL of working reagent (alcoholic solution of neocuproine (0.0075 M), Cu (II) chloride (0.02 M) and ammonium acetate buffer (NH_4_CH_3_CO_2_), at a ratio of 1:1:1 ratio, (v:v:v)) was mixed with 20 µL of samples enriched in sEVs. Each sample was centrifuged at 750 × g for 3 min; the supernatant was carefully collected and its absorbance at 450 nm was determined as a measure of total non-enzymatic antioxidant capacity. The calibration curve was generated using known concentrations of the standard antioxidant Trolox (648471, Merck), a water-soluble analog of α-tocopherol. THIOLs were measured as the concentration of total thiol-reactive enzymatic antioxidants using an automated adaptation of the assay [47] initially described by [48]. The assay is based on the reaction of the thiols present in the sample with 5,5’-dithiobis-(2-nitrobenzoic acid) to form a brightly colored anion with a maximum peak at 412 nm (e412 = 13,600 M^−1^ cm^−1^. A volume of 10 µL of concentrated sEV samples was used for analysis. An automated biochemical analyzer (Olympus AU400 Automatic Chemistry Analyzer, Olympus Europe GmbH; Germany) was used for the two analyses. Three technical replicates per sEV sample were performed, and the intra- and inter-assay variability was less than 15% in both assays. Results are expressed as µmol per mg of total protein.

### Statistical analysis

Statistical analyses were performed using IBM SPSS Ver. 27.0 (IBM Corp., Armonk, NY, USA). First, normal distribution and homogeneity of variances were checked using the Shapiro–Wilk and Levene tests, respectively. Simple linear regression tests were run to obtain the best-fit lines (slope, X-intercept and Y-intercept) using the least squares method. The unpaired t-tests or Mann–Whitney tests were run to compare the characteristics of sEVs between large and small sEV samples, and the total antioxidant capacity between non-permeabilized and permeabilized samples, depending whether samples fitted to a normal distribution or not. A two-way repeated measures ANOVA (factors: sEV subtype and H_2_O_2_ treatment) was used to compare the capacity of non-permeabilized and permeabilized sEV samples to protect against oxidative DNA damage. Multiple comparisons were calculated using the Tukey’s post-hoc test. The level of statistical significance was set at *P* ≤ 0.05.

## Results

Our study followed different experiments to evaluate the protective capacity of sEVs against oxidative DNA damage. First, a cell-free in vitro evaluation method of oxidative SSBs was established using plasmid DNA. The generated SSBs were detected using a modified qPCR, taking advantage of the generated 3’-OH ends as priming sites for the DNA polymerase, and was validated with the TUNEL assay. The experimental approach included testing two different subtypes of sEVs of different size, isolated from porcine seminal plasma, which were permeabilized (IC) or non-permeabilized (EV surface). These sEVs were incubated with dose-dependent concentrations of H_2_O_2_ to determine the protective activity of sEVs against oxidative DNA damage. Finally, an insight on the antioxidant capacity of both sEVs subtypes were assessed.

### Development and validation of a qPCR-based method to assess the generation of SSBs on free DNA

**Fig. 2:**
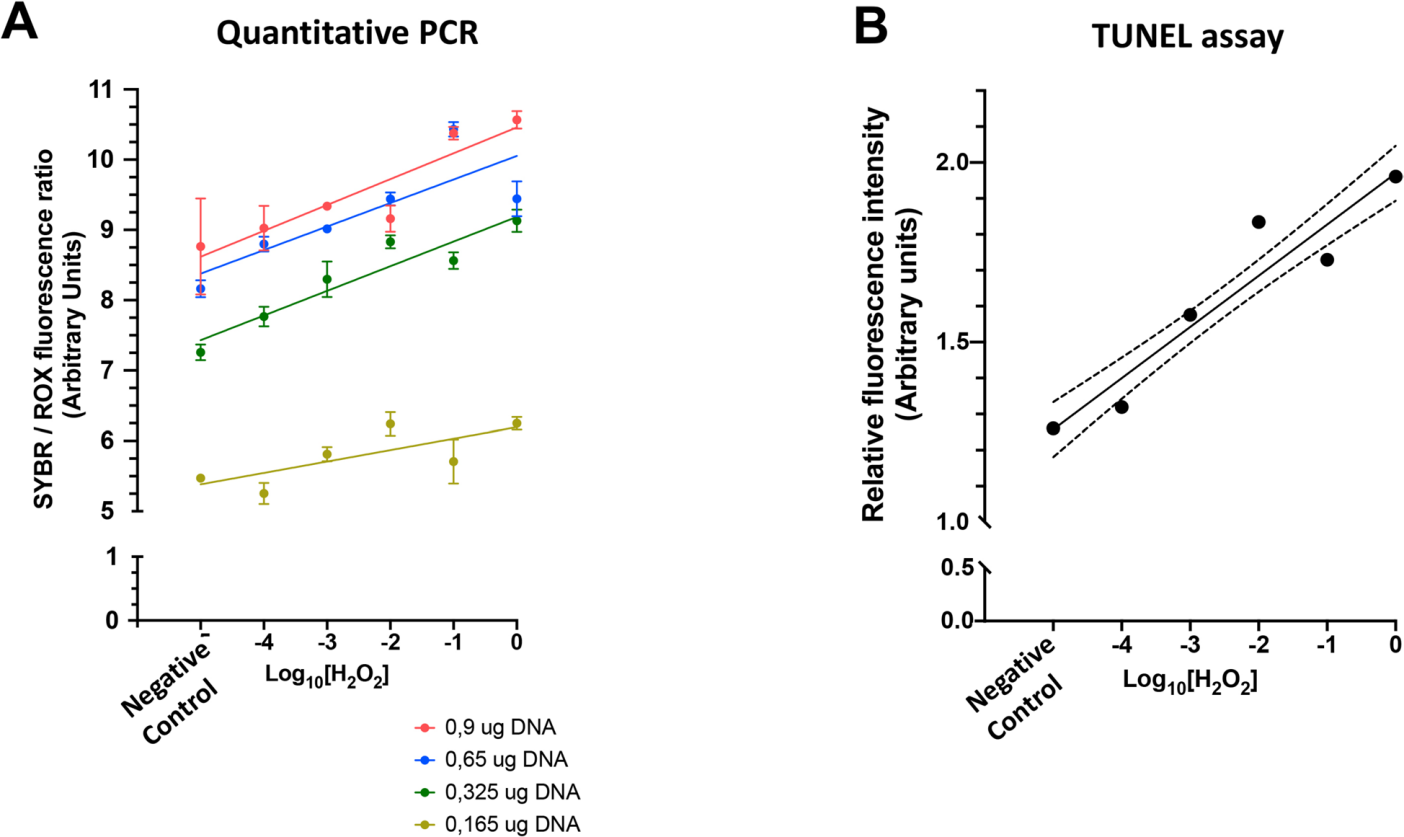
Set up and validation of the method. **A** Dose-dependent generation of DNA single-strand breaks (SSBs) by different concentrations of hydrogen peroxide (H_2_O_2_) detected by assessing separate amounts of DNA in the quantitative PCR-based approach. **B** Validation of the dose-dependent generation of DNA SSBs through the TUNEL assay. The straight line in each figure represents the linear regression equation (Simple linear regression analysis, least squares method). Dotted lines represent the 95% of the confidence interval

First, the pGADT7-T plasmid was transformed into *E. coli* One Shot TOP10 and properly amplified, resulting in a concentration of 1.3 mg/mL and a 260/280 absorbance ratio of 1.875. The agarose gel showed no DNA degradation, as there was no undersized DNA even at high plasmid concentrations (Supplementary Figure S1). This plasmid was then used to interrogate whether the generation of SSB in DNA relied upon H_2_O_2_ in a dose-dependent manner. The experiments were performed using different DNA amounts to set the qPCR conditions. The incubation with increasing H_2_O_2_ concentrations increased the SYBR/ROX fluorescence ratio (Fig. 2A). The slope for this increase was statistically significant for all the H_2_O_2_ concentrations tested (*P* < 0.0001). Remarkably, the linear regression equation fitted better for a DNA concentration of 0.325 μg DNA (R = 0.906; *P* < 0.0001), than for other concentrations (0.900 μg DNA: R = 0.860; *P* < 0.0001; 0.650 μg: R = 0.832; *P* < 0.0001; and 0.165 μg DNA: R = 0.676; *P* < 0.0001). The regression equation when 0.325 μg of DNA was used as a template for the qPCR was linear (Eq. 1, Fig. 2A), indicating that the extent of SSBs in DNA was related to the concentrations of H_2_O_2_ during incubation.

1 $${\text{SYBR }}/{\text{ ROX }} = \, 0.{3}0{7} \times {\text{Log}}_{{{1}0}} \left[ {{\text{H}}_{{2}} {\text{O}}_{{2}} } \right] \, + { 9}.0{38}$$

The relationship between these two parameters (i.e., SYBR/ROS and Log_10_[H_2_O_2_]) was validated by an independent TUNEL assay performed with the same concentrations of H_2_O_2_ (Fig. 2B). The slope of the linear regression equation that related FITC fluorescence intensity to Log_10_[H_2_O_2_] was statistically significant (R = 0.948; *P* < 0.0001; Eq. 2).

2 $${\text{TUNEL FITC intensity }} = \, 0.{143} \times {\text{Log}}_{{{1}0}} \left( {{\text{H}}_{{2}} {\text{O}}_{{2}} } \right) \, + { 1}.{97}0$$

Finally, and in order to mimic the natural concentrations present in the biological system, the use of soluble plasmid DNA to assess the antioxidant activity of sEVs required that the amount of plasmid DNA matched the sperm count. For this purpose, the DNA was extracted from samples with different sperm counts, which led to setting a robust regression equation (R = 0.970; *P* < 0.0001) relating the DNA amount (in ng) with sperm count (in million) (Eq. 3, Supplementary Figure S2).

3 $${\text{DNA quantity }} = { 7}0.{81} \times {\text{Sperm count }}{-}{ 15}.{98}$$

From Eq. 3, 1 µg DNA is equivalent to 14.3 × 10^6^ spermatozoa. This was used to determine the amount of sEV required to test the effects of sEV on oxidative DNA damage in the subsequent experiments.

### Two phenotypically distinct sEV subtypes may be isolated from seminal plasma

**Fig. 3:**
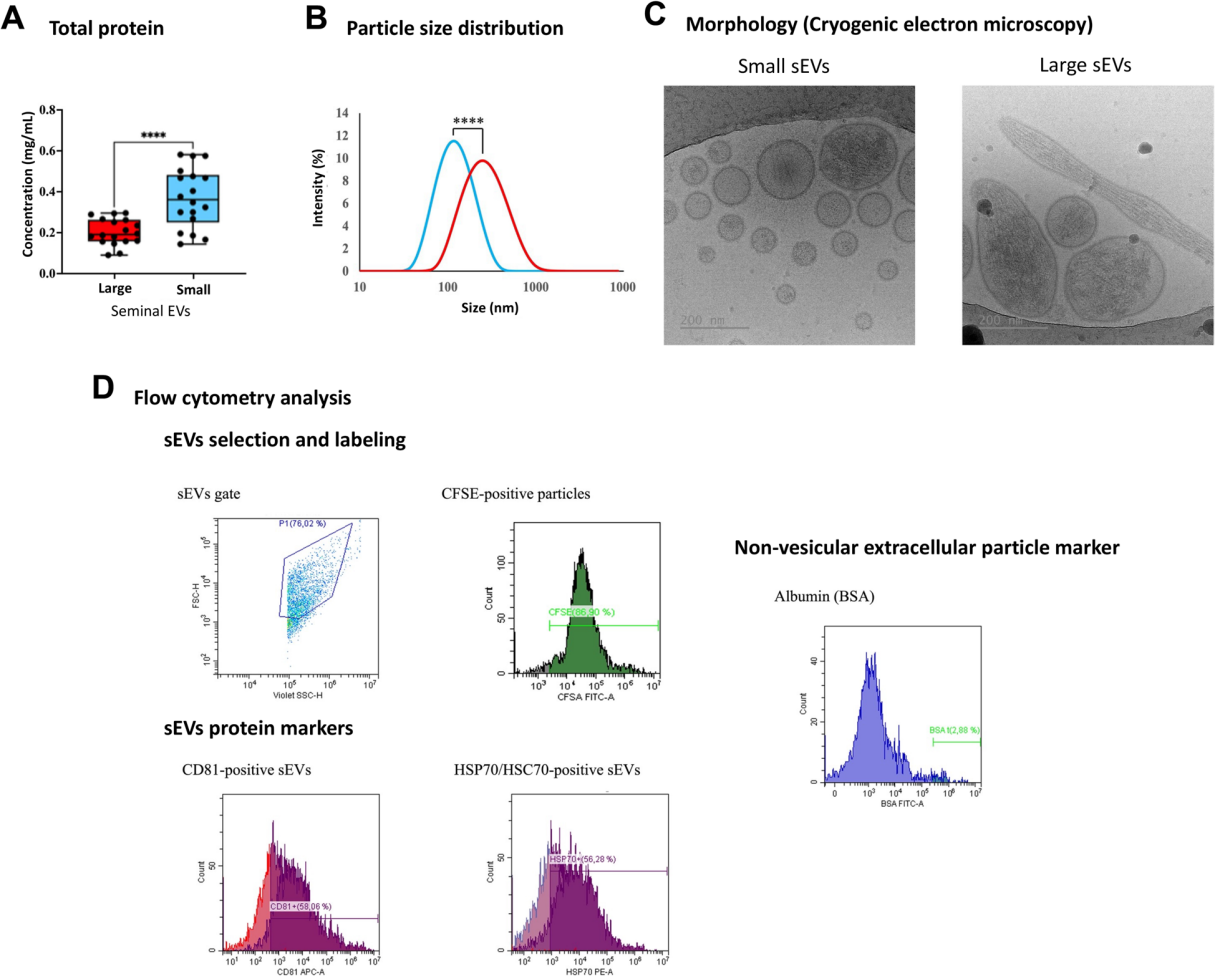
Characterization of small and large extracellular vesicle (EV) samples isolated from porcine seminal plasma. **A** Total protein concentration measured by Nanodrop. **B** Particle size distribution as measured by dynamic light scattering. **C** Representative cryo-electron micrographs showing the morphology of seminal EVs. **D** Representative flow cytometry plots showing the gating of EV and the identification of seminal EVs by labeling with carboxyfluorescein succinimidyl ester (CFSE), EV protein markers (tetraspanin CD81 and cytosolic protein HSP70/HSC70), and a non-vesicular extracellular particle marker (albumin; BSA). (****) show statistically significant differences (Mann–Whitney U test, *P* < 0.0001)

Using a SEC-based protocol, two different size subtypes of sEVs, namely small and large sEVs, were isolated from the seminal plasma. Total protein concentration (mean ± SD) was greater (*P* < 0.001) in small (0.37 ± 0.14 mg/mL) than in large sEV samples (0.20 ± 0.06 mg/mL) (Fig. 3A). The particle size distribution (median and interquartile range, 25th–75th) of the small sEVs samples (133.30 nm; 129.77–136.08 nm) was smaller (*P* < 0.0001) than that of the large sEVs samples (301.63 nm; 284.99–316.71 nm) (Fig. 3B). In addition to size, the two subtypes of sEVs also showed differences in other phenotypic variables. Cryo-EM images, which confirmed the differences in size between small and large sEVs, showed variations in shape between these two subtypes of sEVs. In effect, the small sEVs were mostly rounded, whereas the large ones exhibited a heterogeneous shape, including ovoid and elongated morphologies (Fig. 3C). Flow cytometry revealed that most of the identified nanoparticles were sEVs, as the percentage of positive CFSE was over 75%, notwithstanding this percentage was lower (*P* < 0.01) in small (mean ± SD, 75.26 ± 3.29%) than large sEV (84.83 ± 1.55%) (Fig. 3D). The two sEV subtype samples showed similar percentages of positive events for the two EV-specific protein markers. The percentages (mean ± SD) of events positive for CD81 were 57.27 ± 7.23% and 60.19 ± 2.86% in small and large sEVs, respectively. The percentages (mean ± SD) of HSP70/HSC70 positive events were 55.90 ± 8.99% and 58.05 ± 5.09% in the small and large sEV samples, respectively. Flow cytometry also showed that the percentage of albumin-positive events (mean ± SD) was low in the two sEV subtype samples, although it was higher (*P* < 0.01) in small (3.01 ± 0.39%) than in large sEVs (1.26 ± 0.97%) (Fig. 3D).

### Surface of small seminal EVs exhibits protection against oxidative DNA damage

**Fig. 4:**
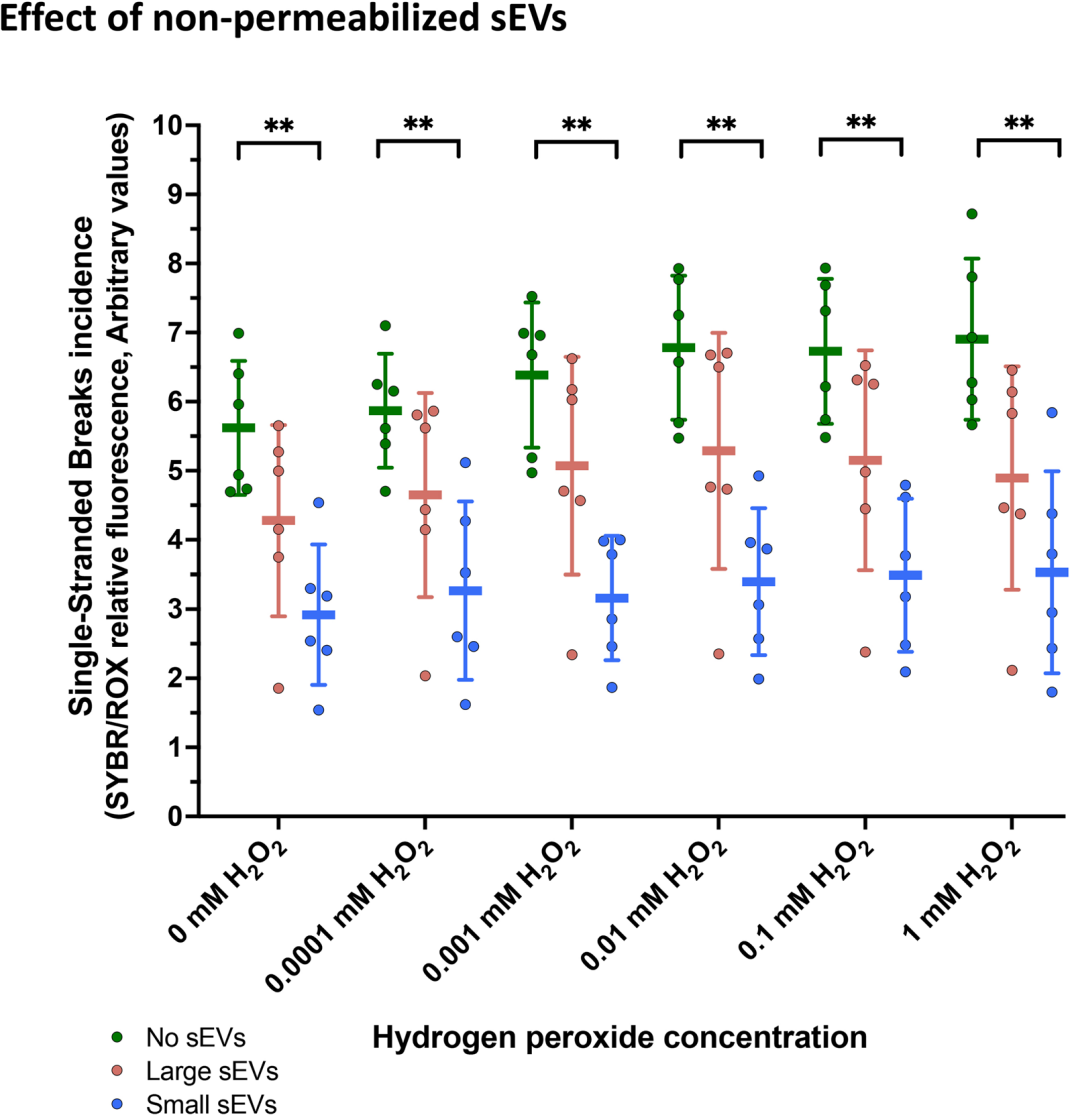
Evaluation of the protective effect of the surface of porcine seminal extracellular vesicles (sEVs) upon oxidative DNA single-stranded breaks generated by different concentrations of hydrogen peroxide (H_2_O_2_). In this experiment, the sEVs were not permeabilized. Green indicates controls without sEVs (control), red indicates co-incubations with large sEV samples, and blue indicates co-incubations with small sEV samples. (**) show statistically significant differences (*P* < 0.01) (Two-way repeated measures ANOVA including sEV subtype and H_2_O_2_ concentration as factors; Tukey’s post-hoc test)

Two experiments were performed to assess the putative role of sEVs in protecting the DNA from oxidative damage; one using non-permeabilized sEVs and the other using permeabilized sEVs. Controls showed that H_2_O_2_ resulted in a dose-dependent increase of the amounts of SSBs in DNA (*P* < 0.01), as observed in the first experiment, validating our model. The first experiment using non-permeabilized small and large sEVs showed that sEV subtypes reduced the extent of SSBs (*P* < 0.01; Fig. 4). Specifically, while the small sEVs mitigated the SSBs induced by H_2_O_2_ at all the tested concentrations (*P* < 0.05, Table 1A), the large ones had no effect on the amount of SSBs (*P* > 0.05, Table 1A). The statistical study of the effect-sizes showed a Cohen’s d of 7.99 ± 6.07 and an effect-size r of 0.91 ± 0.09 between control and small sEVs. Between control and large sEVs, Cohen’s d was 4.74 ± 6.07, and the effect-size r was 0.47 ± 0.66. The protective effect of small sEVs against SSBs was similar when the different H_2_O_2_ concentrations tested were compared (mean ± SD: 48.03% ± 2.2 0%; range: from 44.12% to 50.42%; Supplementary Figure S3).

**Table 1:** Average values of SYBR/ROX ratios (mean ± SD) for each hydrogen peroxide (H_2_O_2_) concentration in experiments containing two porcine seminal extracellular vesicles (sEVs) subtypes (small and large)

	No sEVs	95% Confidence interval	Small sEVs	95% Confidence interval	Large sEVs	95% Confidence interval	P- value (No sEVs vs small sEVs)	P- value (No sEVs vs large sEVs)
A Non-permeabilized sEVs								
0 mM H_2_O_2_	5.62 ± 0.97	(4.77–6.47)	2.92 ± 1.01	(2.03–3.81)	4.28 ± 1.38	(3.07–5.49)	0.004	0.168
0.0001 mM H_2_O_2_	5.87 ± 0.82	(5.15–6.59)	3.27 ± 1.29	(2.14–4.4)	4.65 ± 1.48	(3.36–5.94)	0.013	0.277
0.001 mM H_2_O_2_	6.39 ± 1.05	(5.46–7.31)	3.16 ± 0.90	(2.37–3.95)	5.07 ± 1.57	(3.69–6.45)	0.001	0.226
0.01 mM H_2_O_2_	6.78 ± 1.04	(5.87–7.7)	3.40 ± 1.06	(2.47–4.33)	5.29 ± 1.71	(3.79–6.79)	0.002	0.231
0.1 mM H_2_O_2_	6.73 ± 1.05	(5.81–7.65)	3.49 ± 1.10	(2.52–4.46)	5.15 ± 1.59	(3.76–6.54)	0.004	0.166
1 mM H_2_O_2_	6.90 ± 1.17	(5.88–7.93)	3.53 ± 1.46	(2.25–4.81)	4.90 ± 1.62	(3.48–6.31)	0.013	0.118
B Permeabilized sEVs								
0 mM H_2_O_2_	3.83 ± 0.95	(2.99–4.66)	2.68 ± 1.14	(1.68–3.68)	2.96 ± 1.14	(1.95–3.96)	0.117	0.063
0.0001 mM H_2_O_2_	4.14 ± 1.14	(3.14–5.14)	3.02 ± 1.34	(1.85–4.19)	3.57 ± 1.22	(2.51–4.64)	0.163	0.323
0.001 mM H_2_O_2_	4.38 ± 0.95	(3.55–5.2)	3.08 ± 1.22	(2.02–4.15)	3.69 ± 1.24	(2.6–4.78)	0.086	0.170
0.01 mM H_2_O_2_	4.24 ± 1.24	(3.15–5.32)	3.47 ± 1.57	(2.1–4.85)	3.51 ± 1.42	(2.27–4.75)	0.544	0.324
0.1 mM H_2_O_2_	4.62 ± 0.85	(3.87–5.36)	2.92 ± 1.63	(1.5–4.35)	3.61 ± 1.23	(2.53–4.68)	0.131	0.323
1 mM H_2_O_2_	4.29 ± 1.22	(3.23–5.36)	2.77 ± 1.39	(1.55–3.98)	3.25 ± 1.22	(2.18–4.32)	0.064	0.103

(A) Shows non-permeabilized sEVs, evaluating the functional role of the EV surface. (B) Shows data on permeabilized sEVs (sEVs treated with 0.1% Triton-100 X), evaluating the functional role of the intravesicular content (IC)

**Fig. 5:**
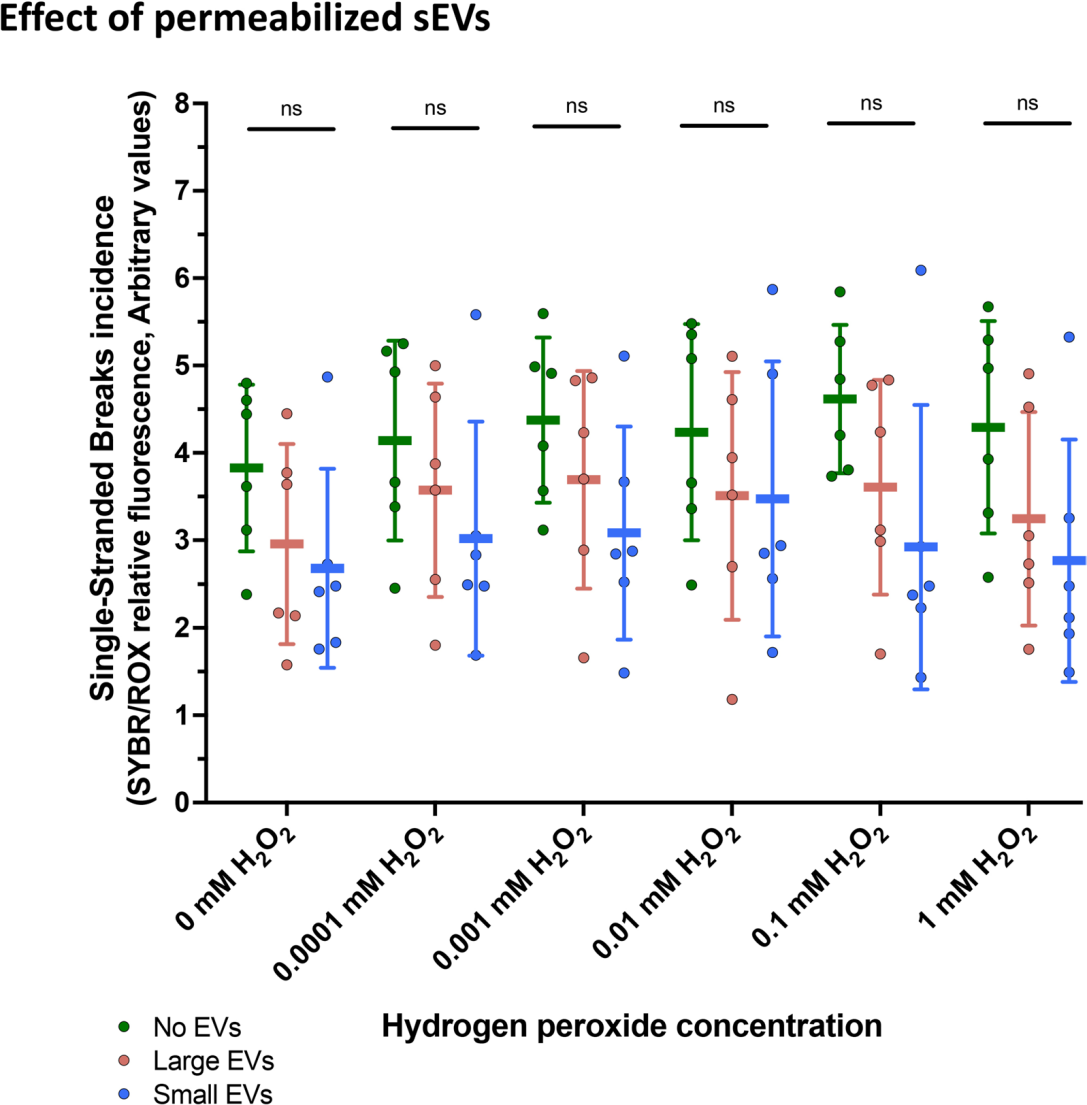
Evaluation of the protective effect of the intravesicular content of porcine seminal extracellular vesicles (sEVs) upon oxidative DNA single-stranded breaks (SSBs) generated by different concentrations of hydrogen peroxide (H_2_O_2_). In this experiment, the sEVs were permeabilized using 0.1% Triton X-100. Green indicates controls without sEVs (control), red indicates co-incubations with large sEV samples, and blue indicates co-incubations with small sEV samples. (n.s.) indicate no significant differences (*P* > 0.05) (Two-way repeated measures ANOVA including sEV subtype and H_2_O_2_ concentration as factors; Tukey’s post-hoc test)

The second experiment comparing permeabilized small and large sEVs showed a similar capability to mitigate the SSBs (P > 0.05) (Fig. 5, Table 1B). The evaluation of the effect-sizes showed a Cohen’s d of 4.18 ± 4.61 and an effect-size r of 0.50 ± 0.57 between control and small sEVs. Between control and large sEVs, Cohen’s d was 2.57 ± 3.48, and the effect-size r was 0.47 ± 0.42.

In summary, the results of these two experiments suggest that the antioxidant capacity of the small sEVs against DNA oxidative damage lies in their EV surface rather than in their IC.

### Enzymatic antioxidant activity in sEVs is localized in the surface of sEVs and is greater in small sEVs than in large sEVs

**Fig. 6:**
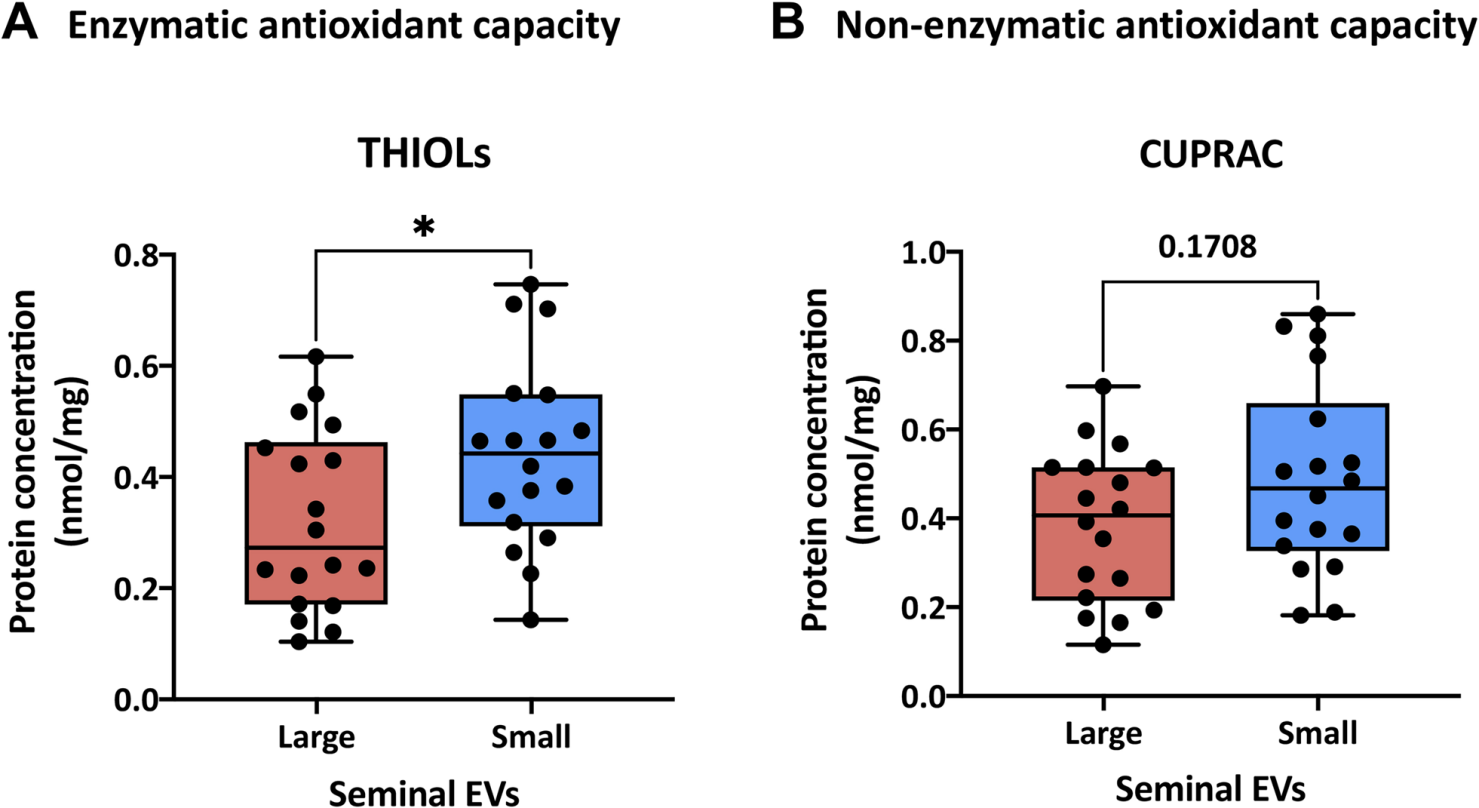
**A** Enzymatic (thiol-reactive antioxidant molecules, THIOLs) and **B** non-enzymatic (cupric ion reducing antioxidant capacity, CUPRAC) antioxidant capacity of small and large porcine seminal extracellular vesicles (sEV). Results of 6 samples of small sEVs and 6 samples of large sEVs, with three technical replicates per each sEV sample. Results are expressed as nmol per mg of total protein. (*) show statistically significant differences (Unpaired T-tests, *P* < 0.05)

Values and confidence intervals for both small and large sEVs antioxidant capacity are shown in Supplementary Table S1. Statistical comparisons showed that antioxidant concentrations were similar between non-permeabilized and permeabilized sEVs (P > 0.05) (Supplementary Figure S4). This indicates that the antioxidant molecules are located on the surface of the sEVs rather than inside these vesicles (IC). Hence, focusing on non-permeabilized sEV samples, the antioxidant content was greater in small than in large sEVs (Fig. 6). Specifically, the differences between small and large sEVs were statistically significant for THIOLs (*P* < 0.05) (Supplementary Table S1).

## Discussion

Extracellular vesicles have emerged as essential players in the regulation of cell functions due to their ability to transport a wide variety of biomolecules from secretory cells to target cells, thus modulating their functional activity [2]. Typically, such modifications contribute to the physiological performance of cells, but in some cases, they contribute to cell dysfunction [49]. The present study evaluated the effect of sEVs on the regulation of redox balance, since OS is very detrimental to cell function as one of the main effectors causing SSBs in DNA [50]. For this purpose, a qPCR-based method was developed to determine the SSBs induced by H_2_O_2_ in plasmid DNA. This method allows for addressing whether the different subtypes of EVs cause or protect the DNA from oxidative damage, avoiding the putative bias associated with the differential uptake of EVs by each cell type.

The proposed qPCR-based method was validated by two different approaches. First, by evidencing that incubations of plasmid DNA with increasing concentrations of H_2_O_2_ caused a dose-dependent increase in SYBR fluorescence. The fluorescence signal is directly related to the amount of SSBs with a free 3’-OH end, which serves as priming site for the synthesis of new DNA strands by DNA polymerases [51]. The second validation was performed by an independent experiment using the TUNEL assay. The TUNEL assay, which directly labels the 3’-OH ends and allows a direct measurement of DNA damage [52], confirmed that the impact of H_2_O_2_ on the induction of that damage was dose-dependent. Hydrogen peroxide has been commonly used to generate SSBs in cell DNA [53], and the SSBs induced by this reagent usually occur in two consecutive steps: oxidation of the DNA bases by the Fenton or Fenton-like reaction, and the enzymatic removal of the oxidized nucleotides, leaving a nick in the DNA [54, 55]. In our method, the plasmid DNA was free from any other biological enzyme, so a free 3’-OH end had to be generated after the oxidation of DNA bases by H_2_O_2_. An additional incubation with the DNA nick-forming enzyme FPG was consequently included in the proposed method [56]. Overall, the developed qPCR-based method was similar to the recently published polymerase-assisted DNA damage assay, which also uses a DNA polymerase to identify SSBs in the alkaline comet assay [57]. As the aim of the study was to interrogate the effects of EVs on the generation or prevention of oxidative DNA damage, the method developed deliberately used soluble DNA and avoided the use of cells. This escaped, among others, the variability associated with EV-cell interactions and related to the cell type. As a result, the method developed can be universally applied to any cell type or EV, making it useful for various purposes.

The choice of EVs isolated from the seminal plasma to validate the method was not baseless. Seminal plasma is rich in EVs, which are highly heterogeneous due to the diversity of origins; in addition, two distinct subtypes of sEVs, which differ in size and composition, can be isolated by SEC with a high degree of purity [37, 44, 58]. Seminal EVs have been suggested to carry many of the functionally active molecules of the seminal plasma, which are involved in regulating sperm function and in modulating the uterine immune environment to facilitate sperm passage and embryo implantation [59–64]. Among other molecules, the seminal plasma is rich in antioxidants, which could also be transported by sEVs [65]. Seminal plasma antioxidants play an essential role in regulating the redox status of spermatozoa, which—despite generating ROS—are poor in antioxidants, and transcriptionally and translationally silent cells [66, 67]. In this context, it is worth noting that the sperm DNA is the most vulnerable target of pro-oxidant molecules [68, 69].

The method developed herein was used to evaluate the protective effect of the two subtypes of sEVs, namely small and large, against oxidative DNA damage. The results showed that small sEVs were more likely to exert a DNA protective effect against oxidative SSBs than were large sEVs. The differences in the composition of these two subtypes of sEVs could explain why small sEVs were more effective, as large and small porcine sEVs vary in terms of protein and lipid content [37, 58].

The protective activity of sEVs against cellular OS has been observed in several diseases. This protective activity could be mediated by the delivery of antioxidant molecules or non-coding RNA to cells, which has generated a mounting interest in the development of new therapies [70, 71]. Particularly in spermatozoa, OS is known to cause genotoxic damage that impairs fertilization and poor embryo development, leading to early pregnancy loss [72–74]. In this regard, a recent study using the mouse as a model found that inducing SSBs in sperm DNA by low doses of H_2_O_2_ reduced embryo development [75]. It is important to note that mature spermatozoa are unable to eliminate the SSBs as the DNA damage repair mechanisms are truncated and no additional gene expression is possible due to the high chromatin condensation [76].

The current study also showed that the protective antioxidant activity of small sEVs resides on their surface, in molecules that are integrated and/or peripherally coupled to the EV membrane. Small sEVs are usually surrounded by a peripheral corona layer (PCL), which consists of proteins and other molecules freely circulating in the body fluid that spontaneously aggregate on the outside of the EV membrane [7, 77]. This is also the case for the sEVs isolated from porcine seminal plasma as shown by cryo-electron microscopy [44]. The PCL of EVs is rich in proteins [11, 78, 79], and recent reports have shown that sEVs contain antioxidant proteins, such as glutathione peroxidase 4 (GPX4), gluthatione S-transferase 1 (GSTP1), superoxide dismutase (SOD), or other proteins that may present antioxidant properties such as CYBRD1, LDHA, LDHB, GSTp1, ATOX1, PRDX1, PRDX2, PRDX5, and ALDH9A1 [37, 80, 81]. The PCL of small sEVs may accumulate antioxidant proteins freely circulating in the seminal plasma. Thus, the PCL of sEVs could protect spermatozoa from OS once bound to the sperm membrane. This protection would be particularly important to prevent the oxidation of sperm DNA. As the high condensation of sperm chromatin does not hinder ROS/RNS from diffusing through protamine-condensed ring structures, these reactive chemical species appear to be the main genotoxic agents [76, 82].

Our results supported that the surface, including the PCL, of small sEVs has more thiols than that of large sEVs. This suggests that the surface of sEVs is loaded with a diverse span of proteins that could exert an antioxidant protection against ROS (O'Flaherty and Scarlata, 2022). In fact, previous studies showed that sEVs are enriched in antioxidant enzymes, suggesting, together with our results that they contribute with ROS scavenging activity that could be key for sperm function and survival [37, 80, 81]. Our study showed a greater abundance of thiols in small sEVs, and this would explain why they have a higher capacity than large sEVs to attenuate the H_2_O_2_-induced DNA damage. The presence of thiols on the surface of sEVs is known to interact with highly reactive compounds, such as ROS, RNS and reactive carbonyl species [83]. Moreover, the thiol-dependent antioxidant system is the primary means of cellular defense against H_2_O_2_, which is, in turn, the major biological ROS [84]. Accordingly, the protective activity provided by the surface of small sEVs would be an important asset in the prevention of oxidative DNA damage in sperm. In this regard, it is important to note that the sperm base excision repair mechanism only contains the enzyme oxoguanine glycosylase (OGG1), which excises the oxidized bases from the DNA strand, creating nicks [85, 86].

**Fig. 7:**
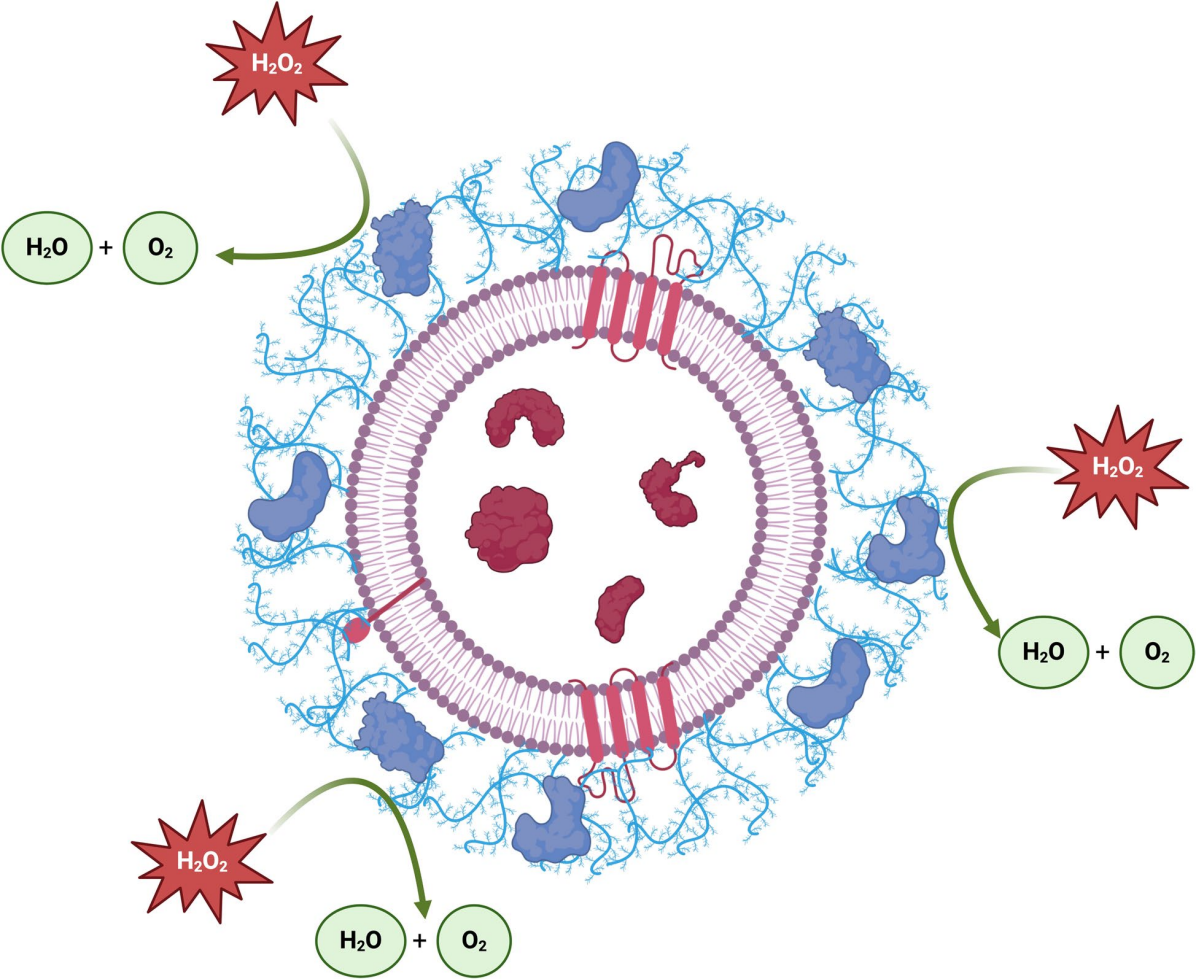
Model showing that molecules located at the surface of small porcine seminal extracellular vesicles are able to counteract oxidative compounds, providing greater protection against oxidation

According to our results, sEVs have the potential to scavenge ROS through enzymatic reactions. Given that, our model depicted in Fig. 7 supports that sEV surface contributes to the redox balance (Fig. 7). The antioxidant microenvironment driven by the surface of small sEVs would supply the ejaculated sperm surrounded by seminal plasma with a better redox regulation and would provide protection to the intravascular compounds that may be useful for sperm function. Overall, this would benefit sperm survival and function. In addition to this, it is now well known that sEVs can bind and integrate into three main regions of spermatozoa, namely the head, midpiece, and flagellum [87]. The head contains the nuclear DNA, which is closer to the external environment, and the midpiece contains the mitochondrial sheath, which is the main source of intrinsic ROS production [69]. Putting together the results from these studies and our work, we hypothesize that if small sEVs were able to become integrated on these regions, they might be able to enrich the sperm plasma membrane with antioxidant enzymes, providing an outer layer capable of scavenging the activity of oxidative molecules and preventing their interaction with the sperm genetic material. However, since our study was not focused to prove that small sEVs caused a change in sperm membrane enzymatic activity, further research should confirm or dismiss this hypothesis.

### Strengths and limitations

Our study has succeeded in developing a method to test the protective activity of EVs against OS in vitro and to differentiate whether this activity is provided by the surface or the IC of small or large EVs. The study, which used EVs from semen, can be extrapolated to EVs derived from any other body fluid or tissue. This confers a broad biological significance to our study, as EVs are widely distributed in the body and are involved in the regulation of many different pathophysiological processes. Despite this achievement, our study is not without limitations. The first limitation would be related to the in vitro nature of the study, which evaluates the protective activity of EVs in solution without considering the interaction of EVs with target cells. Another limitation is that the study used isolated EVs and therefore they do not interact with any other active component of the native fluid that could also be involved in the regulation of OS. For example, seminal plasma contains other soluble antioxidants that could interact synergistically with the effect of sEVs. Future studies should address these limitations and contribute to a deeper understanding of the role of EVs in redox regulation.

## Conclusions

The cell-free DNA damage detection method set herein can test the protective activity of sEVs against genotoxic insults. Moreover, our method has demonstrated that the surface of small sEVs exerts a protective activity against the DNA damage induced by oxidation. This particular protective activity would be mediated by the antioxidant enzymes that are integrated and/or peripherally coupled to the EV membrane.

## Availability of data materials

The datasets used and/or analyzed during the current study are available from the corresponding author on reasonable request.

## Supplementary Information

Additional file 1: Figure S1. Agarose gel containing different amounts of the purified plasmid. No DNA degradation was observed in regions with short DNA length. DNA ladder ranges from 1 to 10 kb.

Additional file 2: Figure S2. Relationship between extracted DNA and sperm count. The straight line represents the linear regression equation, and dotted lines represent the confidence interval.

Additional file 3: Figure S3. Percentage of reduction caused by the co-incubation of non-permeabilized small extracellular vesicles isolated from porcine seminal plasmawith hydrogen peroxidein the experiment evaluating the protective effect of the surface of sEVs. No differences between the tested concentrations of H_2_O_2_ were found.

Additional file 4: Figure S4. Concentrations of non-enzymaticand enzymaticantioxidants in samples of small and large porcine seminal extracellular vesiclessubjected or not to a permeabilization treatment.

Additional file 5: Table S1. Enzymaticandnon-enzymaticantioxidant capacity measured in non-permeabilized and permeabilized seminal extracellular vesicles.

## Data Availability

The datasets used and/or analyzed during the current study are available from the corresponding author on reasonable request.
