# Supplementary figures and images for: Protective role of extracellular vesicles against oxidative DNA damage
Source: Biol Res. 2025 Mar 13;58:14. doi: 10.1186/s40659-025-00595-5 (PMC11905505; doi:10.1186/s40659-025-00595-5)

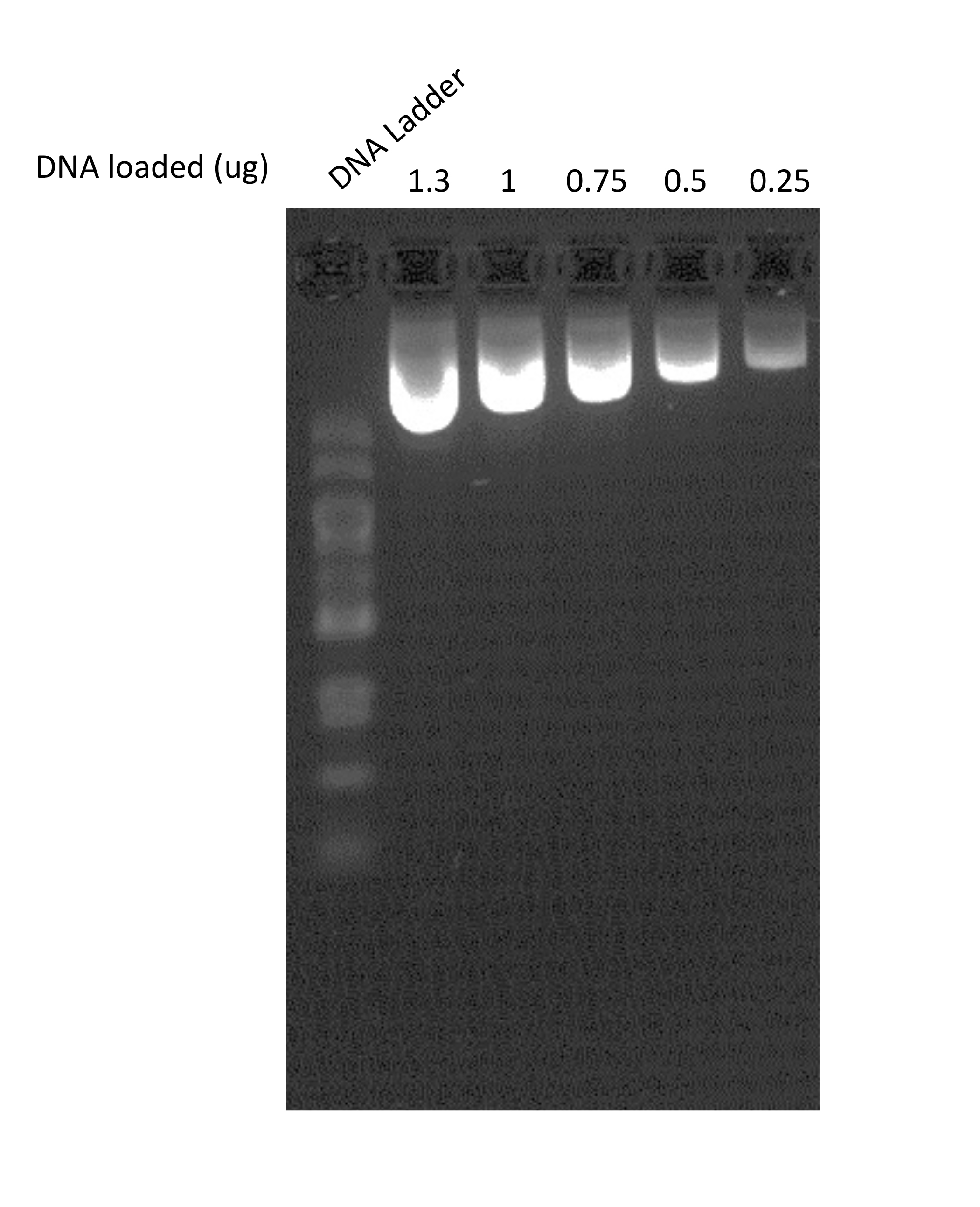

Supplement: Supplementary file 1 — Additional file 1: Figure S1. Agarose gel containing different amounts of the purified plasmid. No DNA degradation was observed in regions with short DNA length. DNA ladder ranges from 1 to 10 kb. [file 40659_2025_595_MOESM1_ESM.jpg]

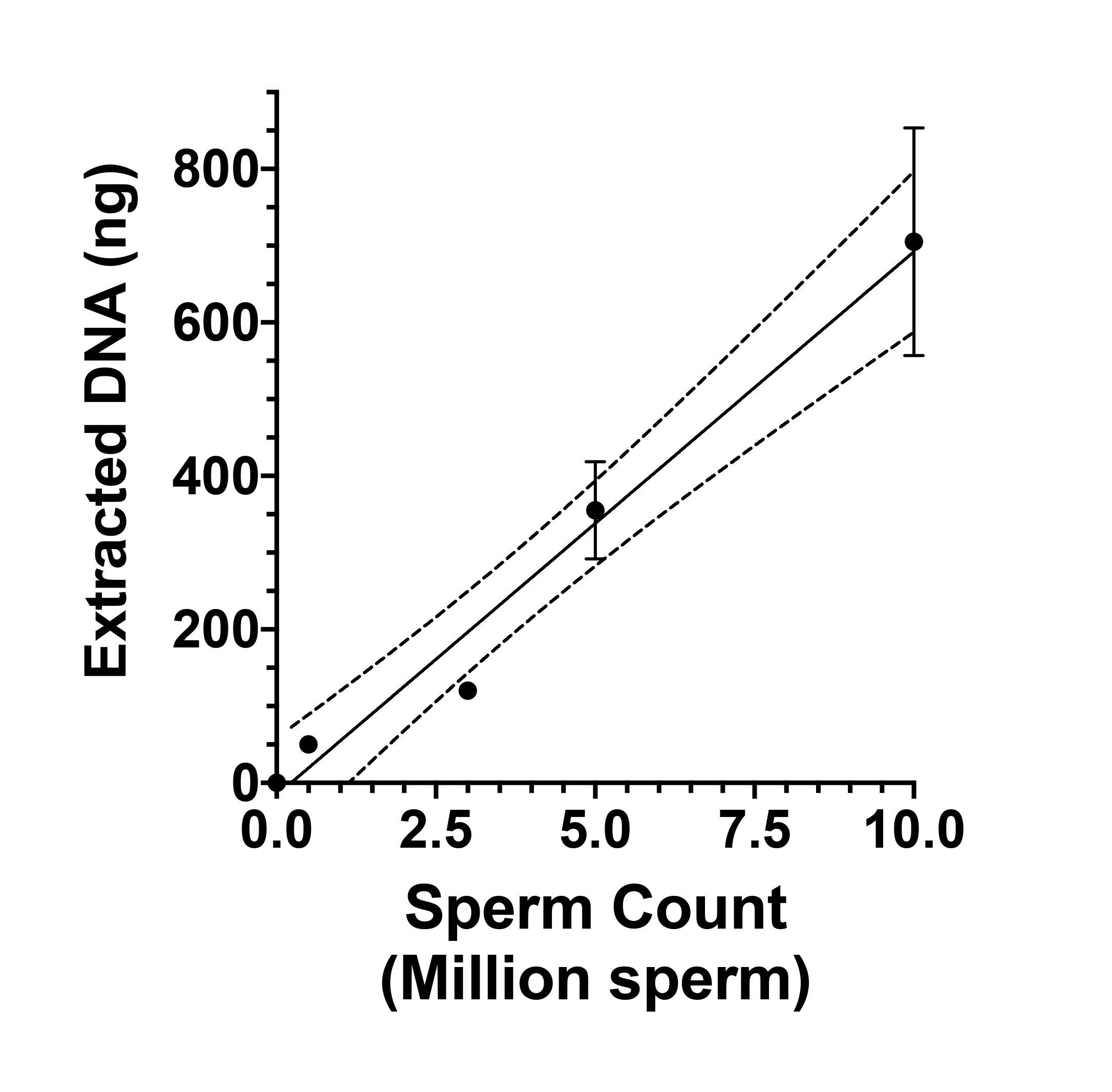

Supplement: Supplementary file 2 — Additional file 2: Figure S2. Relationship between extracted DNA and sperm count. The straight line represents the linear regression equation, and dotted lines represent the confidence interval. [file 40659_2025_595_MOESM2_ESM.jpg]

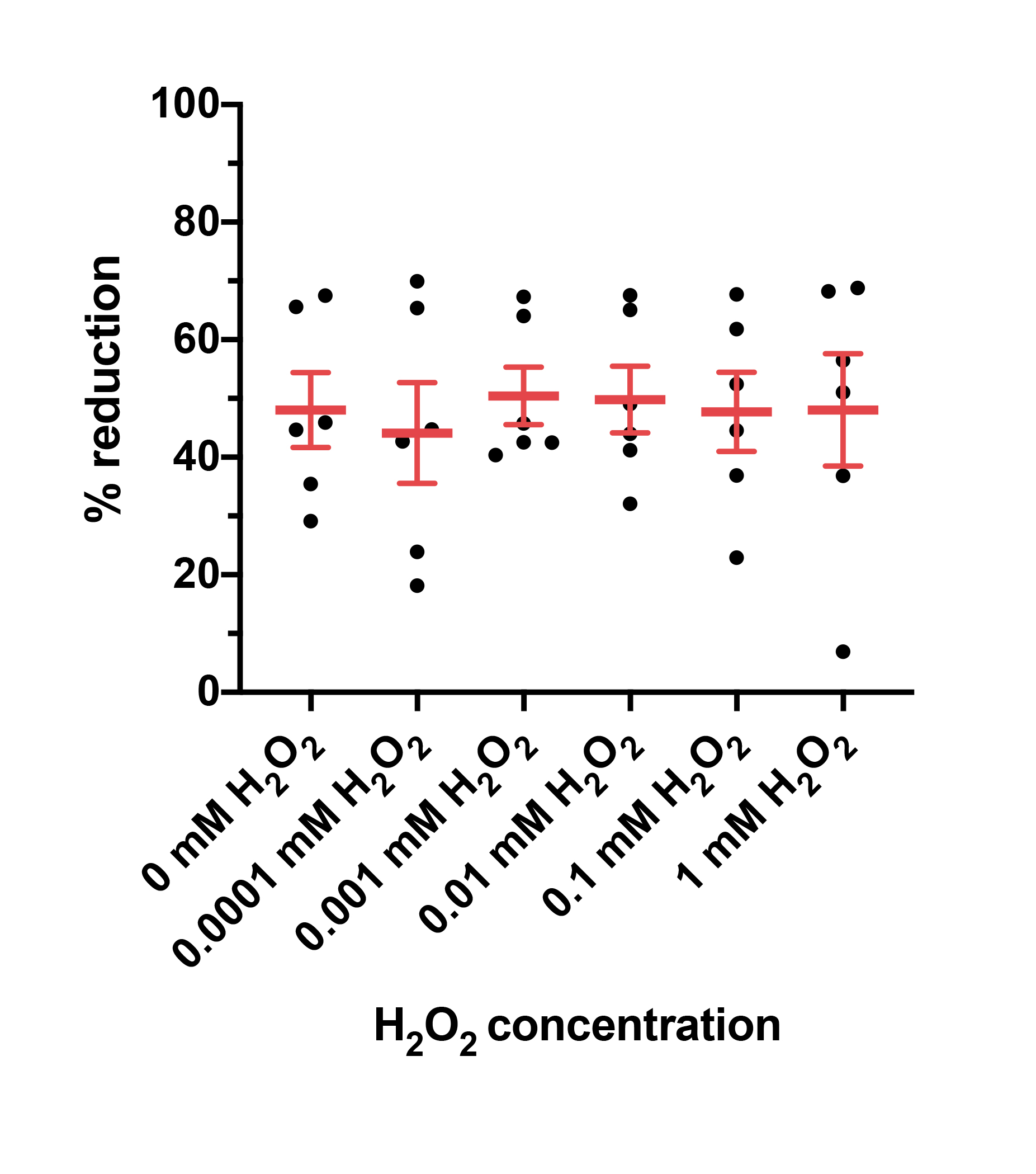

Supplement: Supplementary file 3 — Additional file 3: Figure S3. Percentage of reduction caused by the co-incubation of non-permeabilized small extracellular vesicles isolated from porcine seminal plasmawith hydrogen peroxidein the experiment evaluating the protective effect of the surface of sEVs. No differences between the tested concentrations of H2O2 were found. [file 40659_2025_595_MOESM3_ESM.jpg]

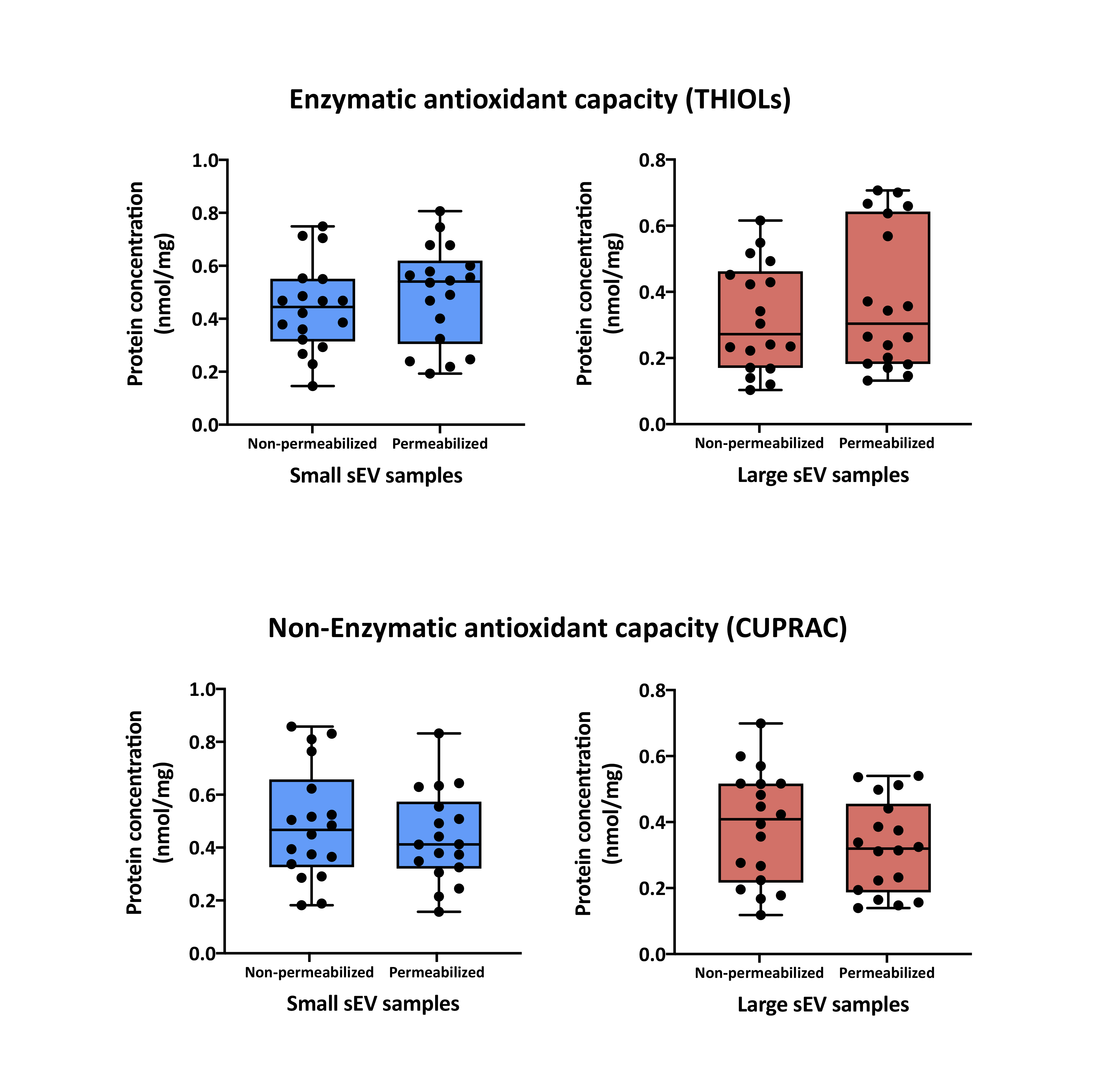

Supplement: Supplementary file 4 — Additional file 4: Figure S4. Concentrations of non-enzymaticand enzymaticantioxidants in samples of small and large porcine seminal extracellular vesiclessubjected or not to a permeabilization treatment. [file 40659_2025_595_MOESM4_ESM.jpg]
