# Supplementary material for: Protective role of extracellular vesicles against oxidative DNA damage
Source: Biol Res. 2025 Mar 13;58:14. doi: 10.1186/s40659-025-00595-5 (PMC11905505; doi:10.1186/s40659-025-00595-5)
Supplement: Supplementary file 5 — Additional file 5: Table S1. Enzymaticandnon-enzymaticantioxidant capacity measured in non-permeabilized and permeabilized seminal extracellular vesicles. [file 40659_2025_595_MOESM5_ESM.docx]

**Supplementary Table S1**. Enzymatic (thiol-reactive antioxidant molecules, THIOLs) and (B) non-enzymatic (cupric ion reducing antioxidant capacity, CUPRAC) antioxidant capacity measured in non-permeabilized and permeabilized seminal extracellular vesicles (sEVs).

| **Enzymatic antioxidant capacity (THIOLs)** | | | | |  | |  | |
| --- | --- | --- | --- | --- | --- | --- | --- | --- |
| *Small sEVs* |  | Protein concentration (nmol/mg) | | |  | | 95% Confidence interval | |
| Non-permeabilized small sEVs |  | 0.47 ± 0.15 * | | |  | | (0.39 – 0.55) | |
| Permeabilized small sEVs |  | 0.5 ± 0.19 | | |  | | (0.4 – 0.6) | |
|  |  |  | | |  | |  | |
| *Large sEVs* |  |  | | |  | |  | |
| Non-permeabilized large sEVs |  | 0.31 ± 0.17 * | | |  | | (0.22 – 0.4) | |
| Permeabilized large sEVs |  | 0.35 ± 0.2 | | |  | | (0.24 – 0.46) | |
|  |  |  | | |  | |  | |
| **Non-enzymatic antioxidant capacity (CUPRAC)** | | | | |  | |  | |
| *Small sEVs* |  | | Protein concentration (nmol/mg) |  | | 95% Confidence interval | |  |
| Non-permeabilized small sEVs |  | | 0.51 ± 0.22 |  | | (0.39 – 0.63) | |  |
| Permeabilized small sEVs |  | | 0.47 ± 0.16 |  | | (0.38 – 0.55) | |  |
|  |  | |  |  | |  | |  |
| *Large sEVs* |  | |  |  | |  | |  |
| Non-permeabilized large sEVs |  | | 0.37 ± 0.18 |  | | (0.28 – 0.47) | |  |
| Permeabilized large sEVs |  | | 0.32 ± 0.15 |  | | (0.24 – 0.39) | |  |
|  |  | |  |  | |  | |  |
| * Statistically significant differences between small and large sEVs (P<0.05) | | | | | | | |  |
